# The VA National TeleNeurology Program implementation: a mixed-methods evaluation guided by RE-AIM framework

**DOI:** 10.3389/frhs.2023.1210197

**Published:** 2023-08-24

**Authors:** Teresa M. Damush, Jayne R. Wilkinson, Holly Martin, Edward J. Miech, Qing Tang, Stanley Taylor, Joanne K. Daggy, Grace Bastin, Robin Islam, Laura J. Myers, Lauren S. Penney, Aditi Narechania, Steve S. Schreiber, Linda S. Williams

**Affiliations:** ^1^ Richard L. Roudebush VAMC HSR&D EXTEND QUERI, Indianapolis, IN, United States; ^2^ Department of Medicine, Indiana University School of Medicine, Indianapolis, IN, United States; ^3^ Regenstrief Institute, Inc., Indianapolis, IN, United States; ^4^ Corporal Michael J Crescenz VAMC, Philadelphia, PA, United States; ^5^ Department of Neurology, Perelman School of Medicine, University of Pennsylvania, Philadelphia, PA, United States; ^6^ Department of Biostatistics and Health Data Science, Indiana University School of Medicine, Indianapolis, IN, United States; ^7^ South Texas Veterans Health Care System, San Antonio, TX, United States; ^8^ Jesse Brown VAMC, Chicago, IL, United States; ^9^ University of Illinois Chicago, Chicago, IL, United States; ^10^ Northwestern University, Chicago, IL, United States; ^11^ Department of Neurology, University of California, Irvine, Irvine, CA, United States; ^12^ Department of Neurology, Indiana University School of Medicine, Indianapolis, IN, United States

**Keywords:** RE-AIM framework, implementation, telehealth, TeleNeurology, rural health

## Abstract

**Introduction:**

The Veteran Affairs (VA) Office of Rural Health (ORH) funded the Veterans Health Administration (VHA) National TeleNeurology Program (NTNP) as an Enterprise-Wide Initiative (EWI). NTNP is an innovative healthcare delivery model designed to fill the patient access gap for outpatient neurological care especially for Veterans residing in rural communities. The specific aim was to apply the RE-AIM framework in a pragmatic evaluation of NTNP services.

**Materials and methods:**

We conducted a prospective implementation evaluation. Guided by the pragmatic application of the RE-AIM framework, we conceptualized a mixed-methods evaluation for key metrics: (1) reach into the Veteran patient population assessed as total NTNP new patient consult volume and total NTNP clinical encounters (new and return); (2) effectiveness through configurational analysis of conditions leading to high Veteran satisfaction and referring providers perceived effectiveness; (3) adoption and implementation by VA sites through site staff and NTNP interviews; (4) implementation success through perceived management, implementation barriers, facilitators, and adaptations and through rapid qualitative analysis of multiple stakeholders’ assessments; and (5) maintenance of NTNP through monitoring quarterly TeleNeurology consultation volume.

**Results:**

NTNP was successfully implemented in 13 VA Medical Centers over 2 years. The total NTNP new patient consult volume in fiscal year 2021 (FY21) was 836 (58% rurally residing); this increased to 1,706 in fiscal year 2022 (FY22) (55% rurally residing). Total (new and follow-up) NTNP clinical encounters were 1,306 in FY21 and 3,730 in FY22. Overall, the sites reported positive experiences with program implementation and perceived that the program was serving Veterans with little access to neurological care. Veterans also reported high satisfaction with the NTNP program. We identified the patient level of perceived excellent teleneurologist–patient communications, reduced need to drive to get care, and that NTNP provided care that the Veteran otherwise could not access as key factors related to high Veteran satisfaction.

**Conclusions:**

The VA NTNP demonstrated substantial reach, adoption, effectiveness, implementation success, and maintenance over the first 2 years of the program. The NTNP was highly acceptable to both the clinical providers making the referrals and the Veterans receiving the referred video care. The pragmatic application of the RE-AIM framework to guide implementation evaluations is appropriate, comprehensive, and recommended for future applications.

## Introduction

There is a national shortage of general neurology physician staff in the Veterans Health Administration (VHA). The fiscal year (FY) 2018 VHA Shortage Occupations Report cites physician staff (medical officer) as the first ranked clinical occupation, and many Veteran Affairs (VA) healthcare systems list neurology among their shortage occupation selections. When VHA access is limited, community referrals are an alternative option if accessible. Since 2014 with the passing of the Veterans Access, Choice and Accountability Act (Choice Act) and the VA MISSION Act (MISSION Act, 2018), Veterans had increased opportunities to receive care from community providers in the private healthcare sector (1). The expenditures for neurological care in the community are staggering, totaling over $27 million in FY18.

With more care options available, VHA developed the Referral Coordination Initiative (RCI) to help Veterans make informed decisions about where to receive their healthcare (1). The RCI teams review Veteran referrals and consults and review VA care options from traditional face-to-face clinic visits at the VA or in the community, VA telehealth visits, or electronic consults (eConsults) by VA specialists who review the patient chart and provide recommendations without the need for the Veteran to drive to a VA location for care (1).

There are 2.8 million rurally residing Veterans enrolled in VA care (2); many of these areas have little to no neurology coverage (both within VA and the community), and access to care is difficult. In a survey of VA facility leadership, over 20% of facilities reported experiencing difficulty with access to community neurology care (3). While VA community referral costs reflect some aspect of this resource need, it is likely that there are missed opportunities and Veterans who simply do not receive this care at all.

The VA Clinical Video Telehealth (CVT) for specialty care, including neurology, has successfully increased Veteran access to specialists. The encounters of all neurology CVT general neurology, as well as specialty clinics [e.g., Parkinson's, amyotropic lateral sclerosis (ALS), epilepsy], have steadily increased over the last few years. However, most of this growth has represented outreach from large VA Medical Centers (VAMCs) to Veterans within that healthcare system, with no nationwide TeleNeurology program providing access to neurology care at more rural systems.

The VHA National TeleNeurology Program (NTNP), funded in 2020 by the VA Office of Rural Health (ORH) as an Enterprise-Wide Initiative (EWI), is an innovative healthcare delivery model designed to fill the patient access gap for outpatient general and specialty neurological care. It harnesses the VHA neurology expertise from urban (VAMCs) to provide access to outpatient neurology care via teleheath for Veterans residing in rural communities in the United States.

### VA NTNP components

• NTNP hub facility: Philadelphia VAMC where the medical director and program administrator are located and key administrative functions occur.
• Target spoke facilities: VAMCs that have no or insufficient neurology services and serve a large proportion (>50%) of rurally residing Veterans (as per the rurality calculator).
• Target site services include video telehealth new patient and follow-up consultations using CVT or directly to the Veterans’ homes [VA Video Connect (VVC)].

To understand the implementation process of the NTNP across the VHA over a 2-year period (FY 2021–2022), we evaluated the NTNP implementation using a pragmatic application of the RE-AIM framework (4): reach, effectiveness, adoption, implementation, and maintenance. According to RE-AIM, implementation goals stride toward widespread adoption of the innovation, reach a large number of people as intended, sustain implementation at affordable costs, and produce replicable, effective outcomes.

Recently, the NTNP reported program effectiveness in improved Veteran patient access to outpatient neurology services with reduced wait times and lower monthly volume of post-implementation Community Care Neurology (CCN) consults in NTNP vs. control sites (5, 6). In this evaluation, we used additional mixed-methods data to further evaluate the reach, effectiveness, adoption, implementation, and maintenance of the NTNP across participating VAMCs in the first 2 years of program implementation.

## Materials and methods

### Design

We conducted a prospective evaluation of the VA NTNP which was implemented in VHA facilities in rural US communities. We used data collected in the first 2 years of NTNP activity, FY2021–2022. Guided by the RE-AIM framework (7, 8), we utilized a mixed-methods evaluation to collect and analyze data in the five RE-AIM domains (see Table 1).

**Table 1:** RE-AIM evaluation measures.

RE-AIM domain	Domain description	RE-AIM outcomes operationalized
Reach	Who is intended to benefit and who actually participates in the NTNP?	TeleNeurology (new and follow-up) encounters in FY21 and FY22
		Rurality proportion of Veterans served by NTNP VAMCs
Effectiveness	Perceived NTNP satisfaction and usefulness to Veterans and site clinical providers	Veteran NTNP satisfaction
		Comparisons in patient satisfaction between NTNP types of visit: VVC vs. CVT
		Site providers’ satisfaction with NTNP
Adoption	Where is NTNP applied and by whom?	VAMCs go live with NTNP
Implementation	Factors impacting NTNP implementation as planned	Implementation barriers and facilitators
		Leadership and teleneurologists perceptions
		Adaptations
Maintenance	Extent NTNP becomes sustained over time	Change in TeleNeurology clinical encounters from 2021 to 2022

### Setting VA National TeleNeurology Program

The Department of Veteran Affairs NTNP was funded by the ORH with start-up activities in FY2020 and the first clinical implementation at a VA facility in October 2021. In FY2021, the NTNP was implemented at 12 VAMCs, and it is currently active in 13 sites with one more joining in FY2022. Thus, these analyses included data from sites active in FY2021 and FY2022 with a site sample of 13 VAMCs from across VHA. See Table 2 for site-level contextual data.

**Table 2:** The National TeleNeurology Program clinical site adoption.

NTNP site	US region	First meeting date	Pre-implementation	Go live date adoption
2	Mid-Atlantic	17 June 2020	124	19 October 2020
1	Northwest	13 July 2020	123	13 November 2020
4	Mid-Atlantic	7 October 2020	118	2 February 2021
3	Mid-Atlantic	16 November 2020	85	9 February 2021
5^a^	North	19 January 2021	21	9 February 2021
6	Mid-Atlantic	23 December 2020	71	4 March 2021
7	Southwest	18 February 2021	49	8 April 2021
9	Northeast	10 November 2020	169	28 April 2021
8	West	24 February 2021	77	12 May 2021
10	West	20 April 2021	35	25 May 2021
11^b^	Southwest	16 November 2020	197	1 June 2021
12	Southeast	22 June 2021	56	17 August 2021
13	Midwest	4 November 2021	75	18 January 2022

Average days to adopt NTNP = 92.3 (SD = 49.7), and range of days were between 21 and 197. Site 1 no longer had neurology access needs in FY22, and therefore TeleNeurology services were no longer utilized. All VAMCs adopted TeleNeurology in FY2021 with the exception of one VAMC. Site 13 adopted TeleNeurology in 2022.

^a^ They did not have activation meetings. One of NTNP's early team members was a former employee at Site 5 so they facilitated implementation through a series of emails over 2–3 weeks.

^b^ Site 11 had several start and stop moments, and they did not meet this entire time; there was likely high-level discussion outside of the activation meetings.

### National TeleNeurology Program

The NTNP provides outpatient neurological evaluation and management through telehealth delivery by video to home (VVC) and video in an outpatient clinic (CVT). Veterans can choose between NTNP and other neurology services for which they may be eligible, including care in the community (CCN) that is paid for by VA. All data analyzed in this project were collected for operational and quality improvement purposes as part of the NTNP ORH evaluation; this project was approved as operational (non-research) by the VA and the Indiana University Institutional Review Board (see signed memo of understanding).

Candidate NTNP sites were identified by examining site neurology FTE, neurology wait times, and patient rurality data. NTNP leadership would then reach out to site leadership to initiate a conversation about interest in NTNP services. Once the process of site exploratory conversations had begun, other sites sometimes self-identified due to difficulties with neurology access.

### Participants

We included multiple key stakeholders involved in the management, delivery, and utilization of the NTNP healthcare services for our evaluation. Table 3 displays the participants’ survey disposition across the 13 VAMCs. Nested within the 13 VAMCs were key stakeholders: primary care clinical providers and staff who referred and utilized NTNP services, Veteran patients who sought outpatient care at the sites and were the recipients of the NTNP services for neurological services, and VAMC site leadership and management who facilitated local NTNP implementation. For Veterans with an NTNP or CCN consult placed, we captured demographics (age, gender, race) and rurality (defined by VHA as urban, rural, or highly rural) from Corporate Data Warehouse (CDW) data.

**Table 3:** NTNP Veteran patient survey disposition by site.

Site	Total *N* %	Call completed *N* %	Attempted three times *N* %	Patient/proxy refused *N* %	Patient died *N* %	Other *N* %
Site 1	50	26	18	5	0	1
	%	52.0%	36.0%	10.0%	0.0%	2.0%
Site 2	64	43	15	4	0	2
	%	67.2%	23.4%	6.3%	0.0%	3.1%
Site 3	42	25	10	2	0	5
	%	59.5%	23.8%	4.8%	0.0%	11.9%
Site 4	32	14	15	1	0	2
	%	43.8%	46.9%	3.1%	0.0%	6.3%
Site 5	90	40	35	8	2	5
	%	44.4%	38.9%	8.9%	2.2%	5.6%
Site 6	29	12	14	2	0	1
	%	41.4%	48.3%	6.9%	0.0%	3.4%
Site 7	116	55	47	4	0	10
	%	47.41%	40.52%	3.45%	0.00%	8.62%
Site 8	33	13	14	5	0	1
	%	39.4%	42.4%	15.2%	0.0%	3.0%
Site 9	16	6	8	0	0	2
	%	37.5%	50.0%	0.0%	0.0%	12.5%
Site 10	59	26	27	6	0	0
	%	44.1%	45.8%	10.2%	0.0%	0.0%
Site 11	31	9	16	5	0	1
	%	29.0%	51.6%	16.1%	0.0%	3.2%
Site 12	66	21	35	8	0	2
	%	31.8%	53.0%	12.1%	0.0%	3.0%
Site 13	20	11	6	2	0	1
	%	55.0%	30.0%	10.0%	0.0%	5.0%
Total	648	301	260	52	2	33
	%	46.5%	40.1%	8.0%	0.3%	5.1%

Veterans who completed an NTNP consult in the first 6 months of NTNP activity at their site were eligible for a patient satisfaction interview. We attempted three calls with each Veteran seen in the first 3 months of program implementation at that NTNP site and a random 50% of those seen in months 4–6.

### Measurement

We operationalized the RE-AIM domains and outcomes as follows in Table 1 based upon Glasgow and colleagues’ pragmatic applications of RE-AIM for healthcare (7).

### Reach metrics and data

We conceptualized *Reach* into the Veteran patient population as the total NTNP new patient completed consult volume, extracted from the VA CDW and as the total (new and follow-up) NTNP clinical encounters extracted from the VHA Support Service Center Capital Assets (VSSC) database. We excluded consultations for neurology procedures from the CCN consult counts, since NTNP does not provide procedural consultation (e.g., electromyography and nerve conduction studies, electroencephalography studies). Some of these procedures were classified as consultations rather than procedures and therefore were eliminated via Structured Query Language (SQL) text string searching and manual review of consultation names and descriptions at all sites.

### Adoption metrics and data

We measured *Adoption* by VA sites during pre-implementation and early implementation phases using notes from weekly pre-implementation meetings between NTNP and site leaders and interviews with key site personnel 3–6 months after initial implementation of the program.

### Effectiveness metrics and data

We used mixed methods to measure NTNP *Effectiveness* from several perspectives.

#### Access

We measured access to care by calculating time in days from the consultation being placed to being scheduled (for all NTNP and CCN consults with a scheduled date) and from consultation placement to completion (for all NTNP and CCN completed consults). We reviewed any consults that remained in pending status from the study time period to determine if they could be classified as either cancelled/discontinued or completed.

#### Satisfaction

Veteran perspectives on satisfaction and experience with NTNP were collected via telephone interviews conducted within 2 weeks of a completed consult. Veterans who completed an NTNP consult in the first 6 months of NTNP activity at their site were eligible for a patient satisfaction interview. We attempted three calls with each Veteran seen in the first 3 months of program implementation at that NTNP site and a random 50% of those seen in 4–6 months. Questions about satisfaction, similarity of the visit to an in-person visit, and likelihood of recommending a TeleNeurology visit were asked. These questions were individually answered using a 7-point Likert scale where a higher score indicated greater satisfaction. The survey included other questions taken from VA telehealth and other telehealth surveys regarding prior telemedicine experiences, technological difficulties, communication with the neurologist providing care, and the ways in which their TeleNeurology experience did or did not improve access to care (9, 10). These telehealth experience questions were scored on a 5-point Likert scale indicating a degree of agreement with the statement, and higher scores indicated a stronger agreement.

Provider satisfaction was assessed with an emailed survey sent within 1 week of completion of the NTNP consult; if the provider had answered an NTNP survey in the preceding 30 days, the current consult was excluded from the provider survey list. Provider surveys were also completed for the first 6 months after program implementation at each site. Overall satisfaction, whether the consult answered their question, and clarity of the consult were rated on a 1–10 scale, and higher scores indicated greater satisfaction.

Staff perceptions of program effectiveness were assessed during early implementation telephone interviews conducted 3–6 months after the initial implementation began. These interviews are described more fully in the Implementation section that follows.

#### Implementation

Similar to our assessment of effectiveness, we used mixed methods to assess implementation in a variety of ways, from multiple stakeholders, and at different time points. For all interviews, we obtained verbal consent, audio-recorded, and transcribed the interviews. For email surveys and interviews, participation was voluntary. The VA REDCap platform was utilized to email and receive survey responses from clinical providers who had placed a NTNP consult.

#### Pre-implementation activities

We participated in all weekly meetings between NTNP leadership and site leadership once a site had determined to implement NTNP. We took meeting notes and reviewed materials shared by the NTNP team including an implementation checklist with updated notes and comments.

#### Early implementation

We assessed each site's initial experiences with program implementation with staff interviews 3–6 months after implementation. We used the RE-AIM framework to guide development of questions for each type of interview participant (8) as well as a core set of questions across participants. We identified key staff at each site, focusing on site clinical leadership (chief of staff, chief of primary care), operational leadership (primary care general practice manager, telehealth manager), schedulers, and primary care providers. Interviews focused on the site's experiences in NTNP adoption and early implementation, any barriers or facilitators to implementation success, and plans for continued use and maintenance of NTNP.

#### NTNP leadership

We interviewed all of the NTNP leadership and the participating teleneurologists at the end of FY2021 to understand their perspectives on the barriers and facilitators of NTNP implementation and program goals.

#### Maintenance

We operationalized maintenance as the continued use of NTNP consultations as assessed by monitoring quarterly TeleNeurology consultation volume over time using CDW data. We also used early implementation site interviews and NTNP leadership interviews to assess perspectives on likelihood of continuing participation (sites) and strategies to support program growth and maintenance (NTNP leadership).

### Evaluation analyses

#### Reach

We obtained quarterly data, including new consultation volume, status, and timing for both NTNP and CCN consults and reported these data back to NTNP as part of our ongoing evaluation. We used Wilcoxon rank-sum tests to compare demographic characteristics of patients with NTNP and CCN consults, excluding patients that had both an NTNP and a CCN consult.

#### Effectiveness

Accessing metrics of the time in days to schedule a consult (date scheduled minus date placed) and completing a consult (date completed minus date placed) between NTNP and CCN consults were compared using the Wilcoxon rank-sum test, excluding patients that had both an NTNP and a CCN consult. We developed Veteran satisfaction scores as the sum of three key questions (overall satisfaction, recommendation to another Veteran, and likeness to a face-to-face visit), the total score ranged from 3 to 21, and higher scores indicated greater satisfaction. We compared satisfaction mean scores between Veterans with an in-home (VVC) vs. an in-clinic (CVT) NTNP visit using the Wilcoxon rank-sum test with a significance of *p* < 0.05. Provider satisfaction was similarly measured by using overall satisfaction ratings (1–10, higher score indicating greater satisfaction).

We used configurational analysis to look across all individual cases and identify the crucial conditions that distinguished Veterans who rated “highest” satisfaction, a score of 7 on a 1–7 scale, from those who did not. Configurational analysis is a mathematical, cross-case approach that applies Boolean algebra, set theory, and logic to identify a “minimal theory,” i.e., the key difference-making conditions that consistently and uniquely explain an outcome of interest (11, 12). Configurational analysis searches for necessary and sufficient conditions for an outcome to appear and can detect causal complexity (when several conditions must appear together for an outcome to occur) as well as equifinality (when multiple pathways lead to the same outcome) (13, 14).

To aid with factor selection, we began by substantively reviewing the complete set of factors and identifying 45 factors with a theoretically plausible connection to the “high Veteran satisfaction” outcome. Next, using this analytic dataset of 45 factors, we applied the “minimally sufficient conditions” (“msc”) function within the R package “cna” (39) to inductively analyze the dataset and identify configurations of conditions with particularly strong connections to the outcome (i.e., high clinic participation). This configurational approach to data reduction has been described in detail earlier (15, 16). To briefly summarize, we considered all one-, two-, and three-condition configurations that met pre-designated thresholds for consistency and coverage. Consistency is a parameter related to how reliably a model yields an outcome and is calculated as the number of cases covered by the model and have the outcome present divided by the total number of cases covered by the model. Coverage is a parameter indicating explanatory power and is calculated as the number of cases covered by the configuration and have the outcome present divided by the total number of cases that have the outcome. We began with a consistency threshold of 100% and a coverage threshold of at least 15% to avoid overfitting. If no configurations met this dual threshold, we iteratively lowered the specified consistency level by 5 points (e.g., from 100% to 95%, etc.) and repeated the process. We continued relaxing the consistency threshold until at least two potential configurations that met the specified consistency and coverage thresholds were noted. We then assessed all configurations that satisfied those thresholds.

Using this approach, we identified a smaller subset of factors to use in the model-developing phase of the analysis. We developed models by iteratively using model-building functions within the “cna” software package in R (39). We assessed models based on their overall consistency and coverage, as well as potential model ambiguity (when competing models explain the outcome equally well based on consistency and coverage scores). After a preliminary model was identified, we optimized coverage by reviewing the condition table to consider additional configurations that met consistency and coverage thresholds for facilities with a higher impact that were not explained by models developed thus far. We repeated this method for satisfaction item “Would you recommend TeleNeurology to other Veterans like yourself?” Our final models met an overall consistency threshold of ≥75%, a coverage threshold of ≥60%, and no model ambiguity. The coincidence analysis package (“cna”) in R (39), R (version 3.5.0), RStudio (version 1.1.383), and Microsoft Excel were used in analyses.

### Rapid qualitative analyses of implementation data

We used rapid qualitative analysis methods to code pre-implementation meeting notes and early implementation interviews, focusing on elements within domains of the RE-AIM framework (17). We synthesized each site's early implementation interviews, combined with Veteran and provider satisfaction data, into a site report to share with NTNP leadership. We used these site-level early implementation reports for comparisons of RE-AIM elements across sites.

## Results

### Reach

**Figure 1:**
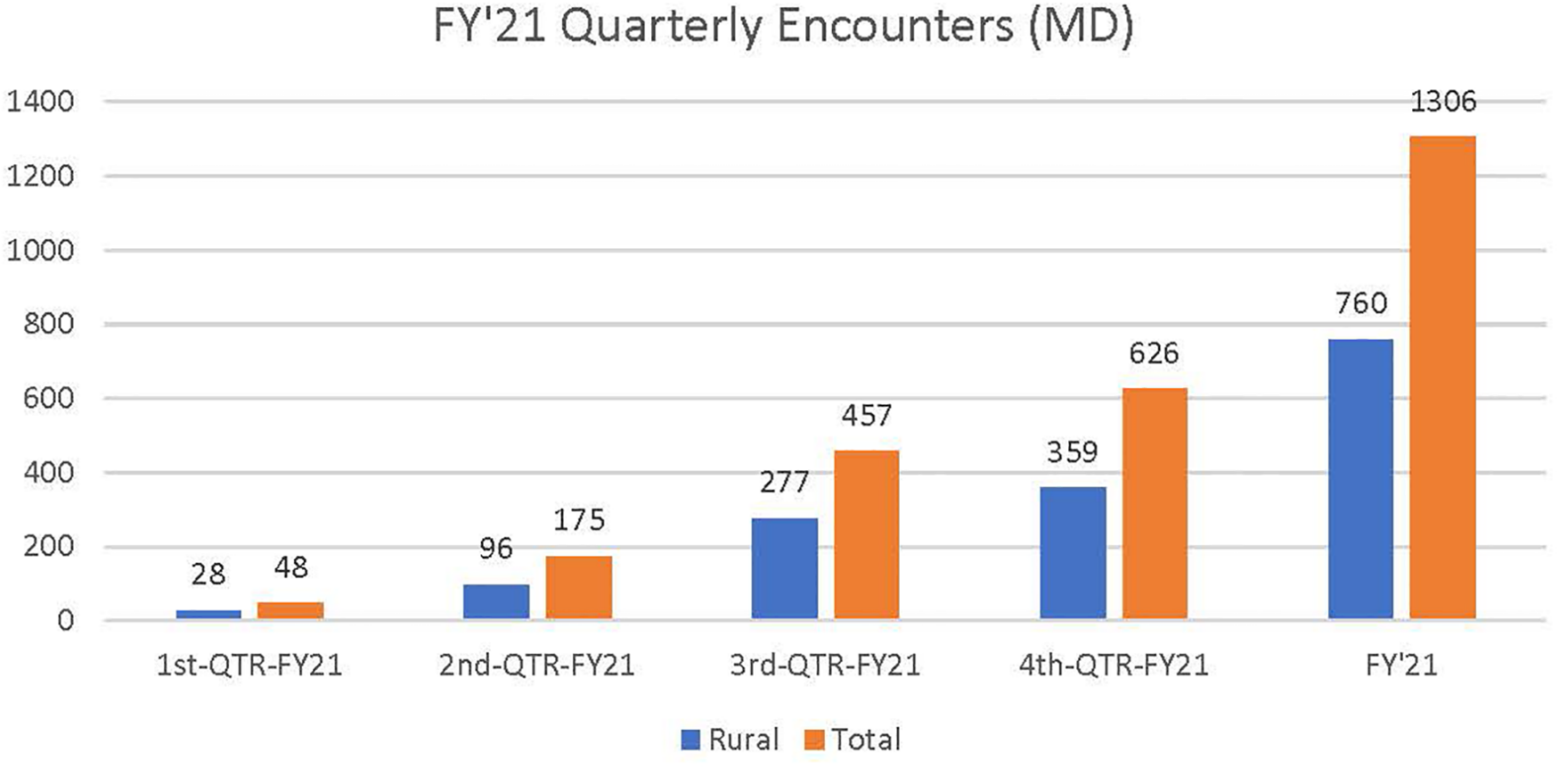
Total NTNP clinical encounters (new and follow-up) in FY 2021. Approximately 58.5% of total NTNP clinical encounters were provided to rural Veteran patients in FY21.

**Figure 2:**
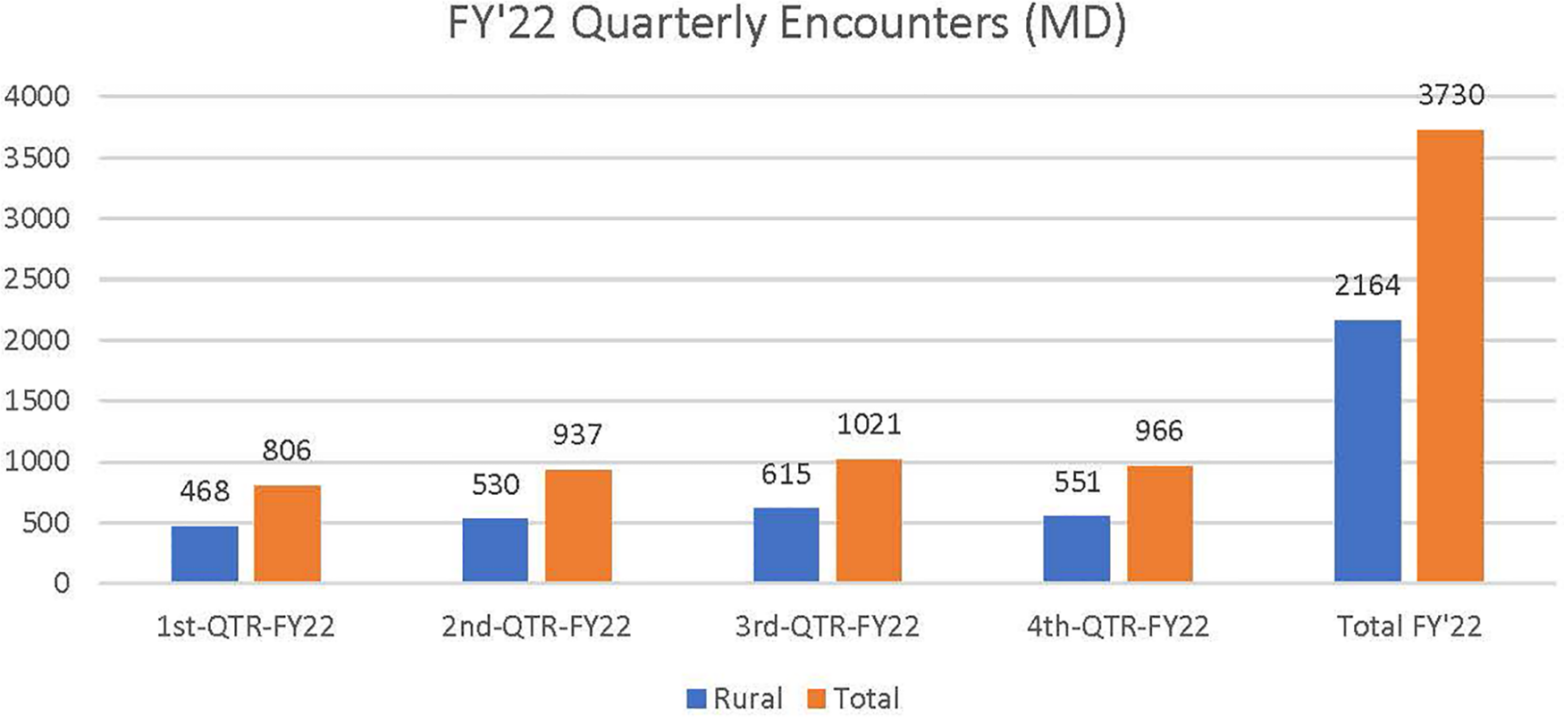
Total NTNP clinical encounters (new and follow-up) in FY 2022. Approximately 59% of total NTNP clinical encounters were provided to rural Veteran patients in FY22.

The total NTNP new patient consult volume was 836 in fiscal year 2021 (FY21) of which 58.1% were rurally residing Veterans and 1,706 in fiscal year 2022 (FY22) of which 55% were rurally residing Veterans. Thus, new patient consults doubled from FY21 to FY22. Total (new and follow-up) NTNP clinical encounters increased almost threefold from 1,306 in FY21 (see Figure 1) to 3,730 in FY22 (see Figure 2).

### Adoption

In FY2021, the NTNP was successfully implemented in 12 VAMCs; six sites had limited local VA outpatient neurology care, and six had none. The time from initial site meeting to program launch averaged 92.3 days, with a range from 21 to 197 days. In FY2022, an additional VAMC (without any local VA outpatient neurology) adopted the NTNP for a total of 13 VAMCs over 2 years in the VHA across the United States with one of those sites halted in the second year since they no longer had neurology access challenges (see Table 3).

### Effectiveness

We conducted Veteran surveys among those who had completed a NTNP virtual visit to determine effectiveness. Of the 648 Veterans eligible, 301 (46.5%) completed a phone interview (see Table 3). Approximately two-thirds of the Veterans surveyed had prior experience with a telehealth visit (see Table 4); approximately half preferred to get their healthcare from the VA, and half preferred to get their healthcare from a mixture of the VA and the community (see Table 5). Almost no Veteran reported that they exclusively preferred community care (0%–8%). Prior telehealth experience varied by site from 38% to 92%. In response to the question, “Why did you choose TeleNeurology service?”, the most frequent responses across the sites were as follows: due to its shorter wait time (35.1%), other reason (35.1%), shorter drive time (22.4%), prefer the VA (4.6%), and perceive VA care to be of higher quality (2.9%) (see Table 6). Overall, sites reported positive experiences with program implementation and that the program was serving Veterans who otherwise would find it difficult to get neurologic care. Veterans also reported high satisfaction with the NTNP program. Mean satisfaction scores (range 1–7) were high across all sites. Combining sites, the overall satisfaction mean score of the program was 6.3 (SD 1.2).

**Table 4:** Patient survey: telehealth experience.

Site	Yes *N* %	No *N* %	Not sure *N* %
Site 1 (*N* = 25)	14	11	0
	56.00%	44.00%	0.00%
Site 2 (*N* = 41)	24	17	0
	58.54%	41.46%	0.00%
Site 3 (*N* = 24)	22	2	0
	91.67%	8.33%	0.00%
Site 4 (*N* = 14)	10	4	0
	71.43%	28.57%	0.00%
Site 5 (*N* = 37)	19	18	0
	51.35%	48.65%	0.00%
Site 6 (*N* = 11)	8	3	0
	72.73%	27.27%	0.00%
Site 7 (*N* = 53)	33	18	2
	62.26%	33.96%	3.77%
Site 8 (*N* = 13)	9	4	0
	69.23%	30.77%	0.00%
Site 9 (*N* = 6)	5	1	0
	83.33%	16.67%	0.00%
Site 10 (*N* = 26)	16	10	0
	61.54%	38.46%	0.00%
Site 11 (*N* = 8)	5	2	1
	62.50%	25.00%	12.50%
Site 12 (*N* = 21)	8	13	0
	38.10%	61.90%	0.00%
Site 13 (*N* = 10)	7	3	0
	70.00%	30.00%	0.00%
Total (289)	180	106	3
	62.28%	36.68%	1.04%

Question: Before your TeleNeurology visit, have you ever used telehealth to interact with a provider before?

**Table 5:** Healthcare preferences.

Site	VA	Non-VA	Prefer a mix
Site 1	9 (39.1%)	1 (4.3%)	13 (56.5%)
Site 2	24 (61.5%)	1 (2.6%)	14 (35.9%)
Site 3	6 (25.0%)	0 (0.0%)	18 (75.0%)
Site 4	6 (42.9%)	0 (0.0%)	8 (57.1%)
Site 5	17 (50.0%)	0 (0.0%)	17 (50.0%)
Site 6	5 (41.7%)	0 (0.0%)	7 (58.3%)
Site 7	21 (42.9%)	4 (8.2%)	24 (49.0%)
Site 8	6 (46.2%)	1 (7.7%)	6 (46.2%)
Site 9	4 (66.7%)	0 (0.0%)	2 (33.3%)
Site 10	14 (53.8%)	0 (0.0%)	12 (46.2%)
Site 11	2 (28.6%)	0 (0.0%)	5 (71.4%)
Site 12	16 (80.0%)	0 (0.0%)	4 (20.0%)
Site 13	4 (44.4%)	0 (0.0%)	5 (55.6%)
Total	134 (48.4%)	7 (2.5%)	136 (49.1%)

Question: Overall, do you prefer to get your healthcare from the VA, outside the VA, or do you prefer a mix?

**Table 6:** Patient survey: main reason.

Site	Prefer VA	Shorter wait time	Shorter drive time	VA care higher quality	Other
Site 1	0 (0.0%)	16 (76.2%)	4 (19.0%)	0 (0.0%)	1 (4.8%)
Site 2	0 (0.0%)	7 (24.1%)	9 (31.0%)	3 (10.3%)	10 (34.5%)
Site 3	3 (15.0%)	5 (25.0%)	6 (30.0%)	0 (0.0%)	6 (30.0%)
Site 4	0 (0.0%)	1 (16.7%)	4 (66.7%)	0 (0.0%)	1 (16.7%)
Site 5	0 (0.0%)	6 (35.3%)	2 (11.8%)	0 (0.0%)	9 (52.9%)
Site 6	1 (12.5%)	1 (12.5%)	2 (25.0%)	0 (0.0%)	4 (50.0%)
Site 7	1 (3.7%)	9 (33.3%)	5 (18.5%)	1 (3.7%)	11 (40.7%)
Site 8	1 (12.5%)	3 (37.5%)	3 (37.5%)	1 (12.5%)	0 (0.0%)
Site 9	0 (0.0%)	1 (25.0%)	1 (25.0%)	0 (0.0%)	2 (50.0%)
Site 10	0 (0.0%)	7 (50.0%)	1 (7.1%)	0 (0.0%)	6 (42.9%)
Site 11	0 (0.0%)	3 (100.0%)	0 (0.0%)	0 (0.0%)	0 (0.0%)
Site 12	2 (22.2%)	1 (11.1%)	1 (11.1%)	0 (0.0%)	5 (55.6%)
Site 13	0 (0.0%)	1 (14.3%)	1 (14.3%)	0 (0.0%)	5 (71.4%)
Total	8 (4.6%)	61 (35.1%)	39 (22.4%)	5 (2.9%)	61 (35.1%)

Question: What is the main reason you chose TeleNeurology for your neurology care?

### Comparison of patient satisfaction by VVC with CVT TeleNeurology visits

In Table 7, we compare the patient satisfaction between the two models of TeleNeurology visits on telepresence (e.g., hear, see, and understand the virtual provider). Overall, Veteran patients attending both NTNP models were satisfied with the telepresence of the visit. However, the Veterans’ ratings of the level of agreement significantly differed on two aspects of the visit. First, Veterans completing a CVT visit reported a higher level of agreement [mean = 4.72 (0.72)] with the statement, “I was able to hear the provider clearly by video,” compared with those completing a VVC visit [mean = 4.45 (0.92)], *p* < 0.02. Second, Veterans who had completed a VVC visit agreed more with the statement that telehealth reduced the need to travel long distances to meet with the provider [mean = 4.69 (0.84)] compared with those who had completed a CVT TeleNeurology visit [mean = 4.36 (1.06)], *p* < 0.02. Veterans who received either a VVC or CVT TeleNeurology visit highly agreed that they could clearly see the provider, could ask the teleneurologist questions, got an appointment on day and time that worked for them, and believed that they received neurological care that they would not have been able to access otherwise.

**Table 7:** Comparisons between VVC and CVT TN visits.

Outcome	VVC Mean (SD)/median (Min, Max)	CVT Mean (SD)/median (Min, Max)	*P*-value (Wilcoxon rank-sum test)
Overall satisfaction (1–7)	*N* = 127	*N* = 116	0.974
	6.28 (1.14)/7 (1, 7)	6.23 (1.24)/7 (1, 7)	
Recommend (1–7)	*N* = 126	*N* = 115	0.872
	6.28 (1.35)/7 (1, 7)	6.30 (1.31)/7 (1, 7)	
Agreement (1–5)			
I was able to clearly see the provider by video	*N* = 121	*N* = 111	0.414
	4.79 (0.60)/5 (1, 5)	4.86 (0.52)/5 (1, 5)	
I was able to clearly hear the provider by video	*N* = 121	*N* = 110	0.020*
	4.45 (0.92)/5 (1, 5)	4.72 (0.72)/5 (1, 5)	
I was able to ask questions directed to the neurologist	*N* = 123	*N* = 110	0.169
	4.89 (0.46)/5 (1, 5)	4.77 (0.62)/5 (1, 5)	
My provider explained things to me in a way that was easy to understand	*N* = 122	*N* = 108	0.479
	4.78 (0.54)/5 (2, 5)	4.69 (0.66)/5 (1, 5)	
My provider listened to me during the appointment in a caring manner	*N* = 121	*N* = 108	0.741
	4.83 (0.57)/5 (1, 5)	4.83 (0.56)/5 (1, 5)	
In general, telehealth reduces the need to travel long distances in order meet with my provider	*N* = 116	*N* = 107	0.018*
	4.69 (0.84)/5 (1, 5)	4.36 (1.06)/5 (1, 5)	
In general, video visits help me get care that I could not access otherwise	*N* = 119	*N* = 102	0.337
	4.33 (1.09)/5 (1, 5)	4.39 (1.12)/5 (1, 5)	
When scheduling my appointment, I was treated with respect	*N* = 116	*N* = 104	0.709
	4.95 (0.32)/5 (2, 5)	4.91 (0.46)/5 (1, 5)	
I got my appointment on a date and time that worked for me	*N* = 118	*N* = 104	0.827
	4.91 (0.47)	4.92 (0.33)	

* *p* < 0.05.

### Qualitative responses

The majority (67%) of the Veterans’ qualitative comments about why they would or would not recommend NTNP were categorized as positive. Most Veterans appreciated the access to care and reduced need to travel provided by telehealth visits:

A great way (Direct telehealth Video to Veteran patient) to communicate; don’t have to travel and can do it on your own time; was a great way to figure out some things and to get some answers; it worked out great and was a good experience. [Veteran patient 521—VVC]

It [TeleNeurology] saved me from driving 100 miles to the next VA …. [Veteran patient 126]

She [Teleneurologist] is the best I’ve ever had. I’ve had other doctors over telehealth, but this was the best. I already recommended her specifically. I would recommend the program because of her [Teleneurologist]. The way she conducted herself, and talked to me and asked me questions, she was not like, ‘Hey, this is your only option,’ she gave me 47 options. And then went through all of them and described whether one was better than the other, etc. She’s very good at what she does. [Veteran patient 651]

Doctor was very caring and interested. Didn’t rush. Wanted to do something for me and wanted to help. [Veteran patient 637]

Negative themes (9%) included a general preference for in-person healthcare and some mistrust around the completeness of the physical assessment via telehealth:

I prefer to be seen in person; I am more of a “hands-on, face-to-face person. [Veteran patient 381]

The doctor had to observe my gait and tremors that I suffer from, and he was doing it over a telephone [video camera]. I think it worked out, I just don’t know how well he was able to capture that from the phone. Definitely would have been able to witness that more in person. [Veteran patient 413]

An additional 24% of Veteran responses about their satisfaction ratings were deemed as neutral. That is, the Veterans expressed both positive and negative comments concurrently often stating the specific encounter was positive, but nonetheless, they prefer in-person visits:

…They (Teleneurologist) treated me with respect, and it was not a troublesome visit at all. I like person to person better, but that’s just me. I didn’t feel like anything was missing from this appointment that would have happened in person, I just think in person it would have been more comfortable for me. But I’m not against telehealth. [Veteran patient 566]

### Configurational analyses

To further understand Veteran factors related to their high satisfaction with NTNP, we conducted configurational analyses. The first configurational model was to identify factors related to Veterans’ rating of overall satisfaction as maximal (score of 7). In this dataset, there were 168 Veterans who reported a 7 vs. 104 Veterans who did not.

The identified model was a single pathway composed of three conditions, all of which had to be present the following: Veterans agreed at the maximum level (five out of five) with the statements that they could ask questions of the teleneurologist, that the teleneurologist listened to them during the video visit, and that telehealth reduced the need to travel long distances for care. This model had 77% (130/168) consistency and 80% (130/162) coverage.

The second model was designed to identify factors related to Veterans’ rating of a maximum score of 7 for the item “Would you recommend TeleNeurology to other Veterans like yourself?” In this dataset, 201 Veterans who reported a 7 for Recommend vs. 85 Veterans for all else were identified.

### This model had two pathways

Veterans agreed at the maximum level (five out of five) that telehealth reduced the need to travel long distances for care, or they agreed at the maximum level (five out of five) with the statement that their provider explained things in a way that was easy to understand and agreed at the second-highest level (four out of five) with the statement that telehealth reduced the need to travel long distances for care. This model had 83% (171/205) consistency and 90% (171/189) coverage.

### Site providers

Clinical providers from the spoke sites who utilized NTNP for their patients had completed surveys about their experiences. The majority of providers (95.8%) reported they would recommend TeleNeurology care for other Veteran patients. Moreover, the clinical providers reported that the NTNP consult addressed their neurologic questions (mean = 9.0, SD = 1.7), the neurologic plan was clear (mean = 9.0, SD = 1.6), and the referring providers were overall satisfied with NTNP services (mean = 8.9, SD = 1.7) (see Table 8).

**Table 8:** Provider consult completion survey.

Site	Consult address mean (SD)	Neurologic plan clear mean (SD)	Overall satisfaction mean (SD)
**Total (148)**	9.0 (1.7)	9.0 (1.6)	8.9 (1.7)
Site 1 (*N* = 14)	7.9 (2.6)	8.9 (1.4)	8.4 (1.8)
Site 2 (*N* = 17)	9.4 (1.3)	9.2 (1.3)	9.3 (1.7)
Site 3 (*N* = 4)	9.5 (1.0)	9.5 (0.6)	9.3 (1.0)
Site 4 (*N* = 9)	10.0 (0.0)	9.7 (0.7)	9.1 (1.7)
Site 5 (*N* = 18)	9.1 (2.1)	8.9 (2.2)	8.9 (2.1)
Site 6 (*N* = 6)	8.5 (2.3)	8.7 (2.1)	8.5 (2.3)
Site 7 (12)	8.8 (1.7)	8.7 (1.8)	8.9 (1.6)
Site 8 (9)	9.3 (0.5)	9.6 (0.5)	9.6 (0.5)
Site 9 (*N* = 6)	9.2 (1.6)	9.0/1.5/2	9.2/1.6/2
Site 10 (13)	8.3 (2.3)	7.6 (2.8)	7.6 (2.8)
Site 11 (*N* = 16)	9.1 (1.5)	9.1 (1.1)	9.1 (1.0)
Site 12 (16)	9.4 (0.9)	9.4 (0.6)	9.3 (0.9)
Site 13 (9)	9.3 (0.9)	9.3 (0.9)	9.3 (0.9)

Likert scale: 1–10, Not at all completely.

Questions:
• How well did the consult address the neurologic question(s) you had about this Veteran? • Did you feel the plan for ongoing neurologic care was clear? • Overall, how satisfied were you with the TeleNeurology program?

### Implementation

NTNP leadership interviews helped identify program aspects that were innovative and viewed as critical to NTNP success, including:

• Establishing the buy-in from the spoke leadership and collaborating with general practice managers.
• Virtually embedding teleneurologists within sites to alleviate inefficiencies of interfacility consults.
• Establishing NTNP as a clinic within the spoke site so teleneurologists directly chart into the spoke site Computerized Patient Record System (CPRS) environment using WebVRAM to improve communication and continuity of care.
• Creating virtual Teams channels for each site and their assigned teleneurologist(s) as a direct and secure communications platform to help “…bring to light problems as well as how to solve [those problems” [NTNP 1].
• Holding NTNP teleneurologists’ team activities like consistent meetings and a journal club, and a clearly shared purpose seemed to positively contribute to a sense of teaming among clinical members. Most expressed strong satisfaction working in the program; one sample quote from a program teleneurologist: “It [NTNP experience] has been wonderful. The whole team is very well connected, everyone is really helpful. The ancillary support as far as connectivity and technology, and anything else we need is there” [NTNP 2].

Challenges noted by NTNP leadership included:

• Attaining site readiness to use the scheduling software, Telehealth Management Platform (TMP), resulting in a shift in initial NTNP implementation focus to providing technological support (e.g., a staff dedicated to training spoke site staff, writing SOPs, and providing feedback to the TMP national team so they could deploy patches as needed).
• Tailoring NTNP to needs at sites where some neurology resources were already available, especially integrating NTNP consult workflow and scheduling into existing neurology consult review and scheduling practices which considerably varied between sites, thus requiring ongoing communication and adaptations at the site level.
• Enhancing site awareness of the availability of the NTNP tools.

The success of the program has helped spur program enhancement in several directions to address identified neurological care resource needs (e.g., number 1 referral for NTNP is for Veterans with headaches). This includes a patient education nurse clinic for headache pilot and neurology brownbag series provided by teleneurologists based on topics chosen by spoke sites. Developments have been pursued due to the enthusiasm of NTNP members; however, moving forward, a caution is to not grow too rapidly and/or overtax staff.

Challenges to implementation included scheduling software utilization, schedulers’ turnover onsite, and variation in the site interpretation of the VA RCI. Facilitators for site implementation included adoption of a uniform scheduling process, coordination of care in real time using Microsoft Teams chat across NTNP, and creation of Teams channels for each site and their assigned teleneurologist(s), establishing NTNP as a clinic within the spoke site so teleneurologists directly chart into the spoke site CPRS environment to improve communication and continuity of care. We also found that NTNP activities including consistent meetings with time allotted for attendance and a journal club and a clearly shared purpose among TN staff seemed to positively contribute to a sense of teaming among clinical members.

Several site adaptations were reported: establishment of a specific RCI process to facilitate NTNP initial implementation and use of “single pathway” neurology consultation to simplify the provider process and ensure that the Veterans are aware of all possible choices for neurology care.

### Maintenance

The NTNP maintenance was evident over time as new visits doubled and total encounters increased threefold from FY21 to FY22. Moreover, FY22 growth was significant both in the clinical and the administrative teams with hiring of over 17 Full Time Equivalent Employee (FTEE) to meet the increased demand in services. The NTNP team now includes 20 active neurologists, seven nurses, one social worker, one clinical pharmacy practitioner, four advanced telehealth technicians, a program manager, an administrative officer, a chief technology officer, a senior teleneurologist–consultant, a lead/supervising neurologist, and a medical director.

All sites in FY22 expressed their intention to continue in NTNP. Facility leadership at the four sites surveyed in FY22 thus far all (100%) indefinitely expect to need NTNP services and are open to some form of cost-sharing with the program in the future. Leadership mentioned Veteran satisfaction, continuing patient/facility demand, and workload credit as being key factors when considering payment for the program.

## Discussion

Our implementation evaluation demonstrated that the VA spoke sites adopted, implemented, and sustained the NTNP video consult services for outpatient neurology care across the VHA targeting VA facilities which serviced Veterans residing in rural communities with limited or no access to local neurology specialty care services either in the VA or out in the community. NTNP had successfully reached into the targeted rural Veteran communities and the VA rural facilities as new patient consults doubled and total clinical encounters tripled over a period of 2 years.

Establishing the local site leadership buy-in during pre-implementation and embedding the teleneurologists’ clinics within the local clinical flow along with establishing virtual platform communications for spoke site clinicians and staff to have direct access to the teleneurologists facilitated the outpatient TeleNeurology implementation. Site readiness to use specific scheduling software and tailoring NTNP workflow to sites with local neurology presented challenges. The innovation of embedding NTNP by setting up virtual local clinics within each site facilitated the implementation and providing specialty care access to rural patients’ enhanced sustainment. Over a 2-year period, NTNP was adopted, implemented, and sustained across rural facilities in VHA. We found executive leadership support and spoke site communications as important facilitators to successful implementation. Indeed, some leadership expressed their willingness to cost-share with NTNP.

The NTNP addresses the VA facility directors’ gaps in specialty care access reported in 2021 (16) by efficiently harnessing the VA neurologists located at urban VA facilities and coordinating their VA virtual outpatient TeleNeurology clinics in rural VA facilities or among sites with limited or no neurology services through telehealthcare delivery. Telehealthcare delivery models redistribute urban resources to the rural patients and communities. Similar to what the virtual cohesive team of teleneurologists built to deliver acute stroke care to VA rural facilities in the VA TeleStroke program (5, 18), NTNP has developed a virtual team of teleneurologists who report high satisfaction with their NTNP organization and with providing VA virtual outpatient neurology services.

Both the spoke sites’ referring providers and the Veteran patients who received NTNP services reported high satisfaction with NTNP. Veterans who received NTNP services at a VA Community-Based Outpatient Clinic (CBOC) through CVT reported similar satisfaction with NTNP compared with Veterans who received direct video telehealth (VVC) except on two areas: Veterans who received CVT reported hearing the providers significantly better than those receiving VVC, and Veterans who received VVC reported that they did not have to drive for miles to see their provider more so than those who received CVT. Our results are in concert with previous research on direct video telehealth visits for outpatient neurology in a single healthcare institution (19) and with CVT for pharmacy outpatient care at VA CBOCs where patients were highly satisfied with CVT visits in comparison with face-to-face visits (20).

The significance of the impacts of the successfully implemented NTNP is enormous. This NTNP telehealth delivery model provided Veteran patients access to acceptable VA specialty care where neurology specialty care both in VA and out in the rural communities (non-VA) were extremely limited or otherwise not available (3). This is important as the Veteran patients who received NTNP services reported that at least 50% preferred to receive VA care and another 50% preferred to receive a mixture of VA and community care. Telehealth virtual delivered care enables the Veteran to receive multidisciplinary guideline concordant care in rural communities. Furthermore, the NTNP services provided the VA RCI located at each VA facility with an additional VA healthcare option for sourcing VA outpatient neurology consults placed by VA primary care clinicians to provide efficient and timely access to high-quality specialty care. Thus, NTNP enhanced the VA RCI coordinator's ability to provide VA care to the Veteran residing in rural communities. This additional VA option for specialty care may be cost-effective for the VHA organization and the Veteran patients. Future research will need to evaluate the cost-savings to determine the cost impacts.

We further examined sets of patient conditions related to high patient NTNP satisfaction using advanced configurational analyses (21). Veterans perceived strong doctor–patient communication skills by the teleneurologists, the reduction of the need to drive long distances for healthcare, and the perceptions that NTNP provided access to neurology care that the patient would not otherwise have as factors related to the highest level of Veteran patient satisfaction with NTNP services.

Similar to other large-scale telehealth program implementation evaluations (22), we applied the RE-AIM framework to guide our NTNP evaluation of the multiple components, reach, effectiveness, adoption, implementation, and maintenance, across the national VHA system. To extend the evaluation beyond the implementation proportions and rates, we intentionally chose to apply the *expanded pragmatic application of RE-AIM* for healthcare by Glasgow and Estabrooks (7). The pragmatic application includes the local context in which adoption, implementation, and maintenance occur, an in-depth evaluation for whom the program reached, which organizations/users implemented the program, implementation barriers and facilitators, and how the program was adapted and maintained. Moreover, our team prospectively extracted deductively RE-AIM elements from qualitative interviews and observations throughout the longitudinal evaluation. We recommend this prospective planning and data extraction to comprehensively capture the framework elements. The results demonstrated the robustness of the pragmatic application of the RE-AIM framework for implementation evaluations, and future comprehensive implementation evaluations should include the expanded, pragmatic RE-AIM application.

## Limitations

Our evaluation of the NTNP had several limitations. First, we limited our sample of Veteran patients who received NTNP services to a site's first 6 months of implementation. Moreover, we reduced the sample by 50% during months 4–6. It is possible that the Veterans’ satisfaction may have changed over time. Nonetheless, we randomized the 50% sample to reduce bias. Furthermore, according to the qualitative analysis, the 9% of Veterans who reported negative perceptions admit that their NTNP experience was positive; however, the negative perceptions were due to the patients’ beliefs that it was necessary to see the clinician face to face for a neurologic visit. Second, our sample of spoke site clinicians was less than 100% of those utilizing NTNP. It is possible that the experiences of the clinician who did not complete a survey or interview differed from those expressed in this evaluation. However, the site clinicians’ evaluations of NTNP were consistently positive across the 13 NTNP sites located across VHA.

## Conclusions

The VA NTNP demonstrated substantial reach, effectiveness, adoption, implementation success, and maintenance over the first 2 years of the program. The NTNP was highly acceptable to both the clinical providers making the referrals and the Veterans receiving the referred video care. Satisfaction with NTNP was related to strong TeleNeurology provider–patient engagement and perceptions that the NTNP provided access to specialty care that Veterans could not otherwise access. The pragmatic application of the RE-AIM framework guided an expanded robust evaluation; therefore, we recommend the pragmatic RE-AIM framework for future applications of implementation evaluations.

## Data Availability

The raw data supporting the conclusions of this article will be made available by the authors, without undue reservation.
